# Choroidal Response to Intravitreal Bevacizumab Injections in Treatment-Naïve Macular Neovascularization Secondary to Chronic Central Serous Chorioretinopathy

**DOI:** 10.3390/biomedicines12122760

**Published:** 2024-12-03

**Authors:** David Rabinovitch, Shiri Shulman, Dafna Goldenberg, Liang Wang, Prashanth Iyer, Anat Loewenstein, Noah Igra, Olivia Levine, Gissel Herrera, Omer Trivizki

**Affiliations:** 1 Tel Aviv Medical Center, Department of Ophthalmology, Tel Aviv University, Tel Aviv 6423906, Israel; 2 Department of Ophthalmology, Bascom Palmer Eye Institute, University of Miami Miller School of Medicine, Miami, FL 33136, USA

**Keywords:** best-corrected visual acuity (BCVA), chronic central serous chorioretinopathy (cCSC), intravitreal bevacizumab (IVB) therapy, macular neovascularization (MNV), optical coherence tomography angiography (OCTA), subfoveal choroidal thickness (SFCT), subretinal fluid (SRF)

## Abstract

Background/Objectives: To evaluate the impact of intravitreal bevacizumab (IVB) therapy on anatomical and visual outcomes in patients with macular neovascularization (MNV) secondary to chronic central serous chorioretinopathy (cCSC). Methods: This retrospective observational study reviewed the medical records of treatment-naïve patients diagnosed with cCSC complicated by MNV and treated with IVB injections over a 5-year period. The presence of MNV was confirmed using optical coherence tomography angiography (OCTA). Best-corrected visual acuity (BCVA), subfoveal choroidal thickness (SFCT), and subretinal fluid (SRF) were recorded pre- and post-IVB treatment. Results: Twenty-two eyes of 22 patients (mean age, 68 ± 11 years) were included. After a mean follow-up of 21.0 ± 14.6 months, SRF significantly decreased from baseline (176.86 ± 115.62 µm) to the final follow-up (80.95 ± 87.32 µm, *p* = 0.003). A greater SRF reduction was associated with more injections (>7) (*p* = 0.047). However, no significant changes were observed in BCVA (*p* > 0.05) or SFCT (*p* > 0.05), irrespective of follow-up duration or injection frequency. Complete resolution of SRF was achieved in nine patients (40.9%), and a significantly greater reduction in SFCT was observed in complete responders compared to non-responders (*p* = 0.03). Conclusions: IVB therapy significantly reduced SRF in cCSC patients with secondary MNV, though it did not lead to visual improvement or significant changes in SFCT. However, greater choroidal thinning in patients with complete fluid resorption may suggest distinct underlying mechanisms or alternative sources of subretinal fluid beyond the MNV itself.

## 1. Introduction

Central serous chorioretinopathy (CSC) is a chorioretinal disease characterized by serous detachment of the neurosensory retina from the retinal pigment epithelium (RPE) and the accumulation of subretinal fluid (SRF) [1]. CSC is generally self-limiting, and approximately 30% of acute cases progress to chronic CSC (cCSC) where persistent SRF can cause diffuse and irreversible damage to the RPE and photoreceptors [2]. In patients with cCSC, up to 39% develop macular neovascularization (MNV), a vision-threatening complication [3,4,5].

CSC is recognized as part of the pachychoroid disease spectrum (PDS), a group of conditions thought to share certain pathogenic features such as thickened choroids and choroidal vascular abnormalities that lead to increased choroidal permeability, RPE damage, and serous (pigment epithelial detachments) PEDs [1,6] However, recent insights sparked by Spaide et al. (2022) suggest that choroidal venous congestion might act as the key driver of CSC pathophysiology [7] In support of this theory, recent advances in multimodal imaging have identified several characteristics of the disease process including choroidal dilation, intervortex venous anastomoses, choroidal hyperpermeability, and RPE dysfunction [8,9] These changes are believed to create the ischemic environment that results in MNV development.

Since the Minerva study [10], which demonstrated the efficacy of ranibizumab in treating MNV across several conditions including CSC, anti-vascular endothelial growth factor (anti-VEGF) VEGF therapy has become the standard treatment for MNV secondary to CSC. However, the efficacy of anti-VEGF monotherapy varies widely, and reliable predictors of treatment success remain elusive [6,10,11,12,13]. Therefore, in patients with MNV secondary to CSC, it is unclear whether SRF accumulation and persistence are primarily due to active neovascularization or the choroidal dysfunction inherent to CSC [14,15,16].

In neovascular age-related macular degeneration (AMD), assessing choroidal changes following anti-VEGF therapy through structural optical coherence tomography (OCT) has provided key insights into disease pathophysiology and has helped optimize treatment strategies [17]. However, in contrast to AMD, where anti-VEGF therapy effectively targets the neovascular process, its role in neovascular cCSC is less clear due to variable and often incomplete treatment responses [10,11,12,13], raising questions about whether fluid accumulation is primarily neovascular or a consequence of CSC-specific choroidal dysfunction. Therefore, understanding of the choroidal response to anti-VEGF treatment in neovascular CSC may similarly elucidate underlying disease mechanisms and enable better stratification of patients most likely to benefit from therapy.

Given the variable efficacy of anti-VEGF monotherapy in neovascular cCSC patients, our objective was to evaluate the visual and anatomical effects of intravitreal bevacizumab (IVB) therapy and to compare the choroidal changes between complete responders and non-responders. These findings may offer insights into the physiological factors underlying treatment response in this condition.

## 2. Materials and Methods

This observational retrospective study was approved by the Ethics Committee of the Assuta Medical Center, Israel. The need for informed consent was waived because of the retrospective nature of the study. All the study protocols adhered to the tenets of the Declaration of Helsinki.

### 2.1. Study Setting, Duration, and Participant Recruitment

The authors retrospectively reviewed clinical records and imaging data from the Ophthalmology Institute at Assuta Medical Center, Tel Aviv, to identify patients with treatment-naïve MNV secondary to cCSC between January 2015 and December 2019.

The inclusion criteria for this study were as follows: (1) patients aged ≥18 years; (2) diagnosis of cCSC as defined by previously published criteria (persistent subretinal fluid [SRF] for ≥6 months) [18]; (3) presence of MNV confirmed on OCT angiography (OCTA); (4) MNV was treatment-naïve at diagnosis; (5) received ≥1 intravitreal injection of bevacizumab (IVB) during follow-up; and (6) had ≥3 months of follow-up.

The exclusion criteria included the following: (1) eyes with a history of other chorioretinal diseases (e.g., diabetic retinopathy, age-related macular degeneration [AMD], retinal arterial or venous occlusive disease, hereditary retinal disease); (2) refractive error greater than ±3 diopters; (3) significant media opacity; (4) pathologic myopia (defined as ≥6.0 diopters); (5) presence of drusen in either eye; (6) neovascularization due to causes other than cCSC; (7) history of retinal surgery within the past 6 months; (8) prior photodynamic therapy (PDT) or laser treatment within the past 6 months; and (9) prior anti-VEGF therapy within 3 months before study enrollment to avoid confounding effects. A total of 28 patients were retrospectively identified as eligible for screening. Following data cleansing procedures, approximately 4.3% to 4.5% of the data points across various follow-up periods were excluded. Ultimately, 22 patients met the eligibility criteria and were included in the study.

### 2.2. Data Collection

Demographic and clinical data, including age, sex, ocular and non-ocular medical history, dates of first cCSC and MNV diagnoses, duration of follow-up, and IVB injection dates and frequency, were retrospectively collected from patient charts. Ocular examination findings were recorded, including best-corrected visual acuity (BCVA), anterior segment examination, and dilated fundus examination. BCVA was evaluated using a Snellen chart and was subsequently converted into the logarithm of the minimal angle of resolution (LogMAR) for statistical analysis.

**Figure 1:**
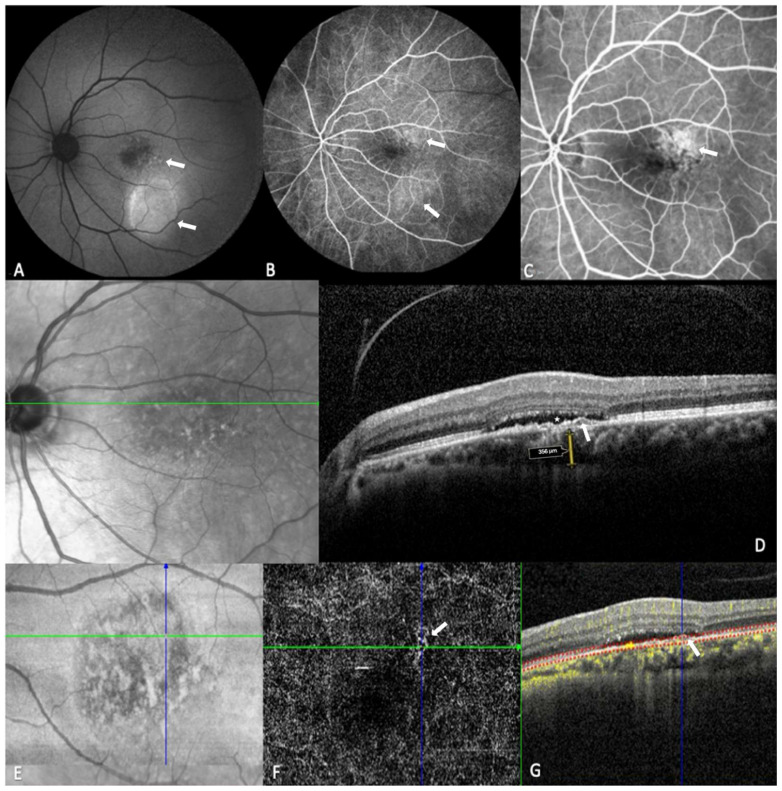
Multimodal imaging of central serous chorioretinopathy (CSC) complicated by type 1 macular neovascularization (MNV) in the left eye, showing correlations among fundus autofluorescence (FAF), fluorescein angiography (FA), optical coherence tomography (OCT), and OCT angiography (OCTA) (**A**–**G**). (**A**) FAF demonstrates hypoautofluorescent changes surrounding the pigment epithelial detachment (PED) (white arrows). (**B**,**C**) FA reveals fluorescein leakage and retinal pigment epithelium (RPE) alterations at 30 s (**B**, white arrows) and 44 s (**C**, white arrow), respectively. (**D**) Structural OCT shows subretinal fluid (SRF) (white star), photoreceptor outer segment atrophy, choroidal thickening (subfoveal choroidal thickness = 356 μm, yellow two-way arrow), and a flat irregular pigment epithelial detachment (FIPED) (white arrow), consistent with type 1 MNV. (**E**,**F**) En face angiography highlights a vascular network corresponding to the FIPED on structural OCT, confirming the presence of type 1 MNV. (**G**) OCT-A (B-scan) demonstrates increased choroidal flow in the region of the MNV (white arrow).

All patients underwent spectral domain OCT (SD-OCT) at baseline and follow-up imaging, with qualitative clinical features such as intraretinal fluid (IRF), SRF, pigment epithelial detachments (PED), and subretinal hyperreflective material (SHRM) evaluated [5,16,17,19,20,21,22]. MNV was confirmed using OCT angiography (OCTA) with Spectralis OCT (Heidelberg Engineering, Heidelberg, Germany). Comprehensive multimodal imaging, including fundus autofluorescence (FAF), fluorescein angiography (FA), optical coherence tomography (OCT), and OCT angiography (OCTA), was evaluated where available (Figure 1).

### 2.3. Image Acquisition and Analysis

Central macular thickness (CMT) was automatically determined using built-in OCT software (version 4.00) and was defined as the distance between the inner RPE border and the inner retinal surface. Using SD-OCT scans, the manual SRF greatest linear dimension and choroidal thickness were measured using an application caliper. Choroidal thickness (CT) measurements were taken at the fovea (for subfoveal choroidal thickness [SFCT]) and measured from the outer border of the RPE to the inner scleral border at 500-micron intervals of a horizontal section from 3 mm nasal and 3 mm temporal to the center of the fovea on EDI scans to account for variation in thickness [23]. Choroidal thickness measurements were consistently performed between 8:00 and 10:00 a.m. to minimize diurnal variations [8]. Choroidal thickness, CMT, and SRF measurements were obtained (1) at presentation pre-treatment, (2) post-treatment after the first set of three IVB injections, and (3) at the final follow-up visit. Measurements were collected by four independent retinal specialists (D.G., S.S., P.I., and O.T.). Scans were mixed and randomized to prevent researchers from knowing the study’s purpose and the pre- and post-treatment imaging conditions.

On OCTA, retinal and choroidal blood flow were assessed by four independent experienced OCTA readers (D.G., S.S., P.I., and O.T.). The choriocapillaris was set 20 μm below the Bruch’s membrane complex of the RPE. To identify the MNV network and confirm the diagnosis, only data with a signal strength index greater than 60 were analyzed. MNV was identified as abnormal flow in the choroidal neovascular network. Patients were identified with type I MNV using Bruch’s membrane-RPE slab, while an outer retina to choriocapillaris (ORCC) slab analysis (spanning the outer retina to the choriocapillaris) was used to identify mixed type 1 and 2 MNV.

### 2.4. Anti-VEGF Treatment

Patients received an initial regimen of 3 monthly intravitreal bevacizumab injections (IVB) (1.25 mg/0.05 mL) and were subsequently managed on a treat-and-extend protocol based on treatment response, with a maximum interval of 6 months between injections.

### 2.5. Outcome Measures

The main outcome measures of this study included: (1) changes in BCVA, SRF, CMT, and SFCT following IVB therapy; and (2) the differences in baseline and follow-up SFCT between complete responders (those with full SRF resorption) and non-responders.

### 2.6. Statistical Analysis

Patient demographics, clinical characteristics, and treatment outcomes were analyzed using Python (version 3.7.9). Changes in BCVA, CMT, SRF, and choroidal thickness over time were assessed using one-way repeated measures analysis of variance (ANOVA), with the Greenhouse–Geisser correction applied for violations of sphericity. A mixed-effects model (REML) was employed to account for time and inter-patient variability, recognizing the inherent heterogeneity in follow-up duration and treatment frequency in this retrospective cohort.

The impact of injection frequency on these parameters was evaluated using regression analysis, adjusting for inter-patient variability and injection intervals. Data normality was confirmed via Q–Q plots, and outliers were identified and excluded using the ROUT method with a 1% false discovery rate, ensuring robustness in findings despite the small sample sizes in specific datasets. Post hoc power analysis was conducted to ensure the adequacy of the methodological approach, yielding power values of 0.85, 0.79, and 0.96 for subfoveal, nasal, and temporal thickness measurements, respectively.

## 3. Results

### 3.1. Baseline Characteristics

The demographic and clinical characteristics of patients are shown in Table 1. The study included 22 patients with MNV secondary to chronic CSC (mean age 67.61 ± 10.65 years, 82% male). The mean duration from the diagnosis of MNV to the first injection was 16.35 ± 13.80 days. At presentation, the mean logMAR visual acuity (VA) was 0.51 ± 0.47. The total follow-up period averaged 21.0 ± 14.6 months. At the initial visit and before starting IVB treatment, 90% of patients had current serous retinal detachments, and 10% had evidence of prior serous retinal detachment on SD-OCT. IRF was found in 50%. Additionally, all patients had at least one area of RPE alteration on SD-OCT, and FIPED was identified in 55%. Type 1 MNV was identified in 11 (50%) patients using a Bruch’s membrane-RPE slab, while the remaining 11 (50%) had mixed type 1 and 2 MNV as determined using outer retina to choriocapillaris (ORCC) slab analysis.

### 3.2. Anatomic and Visual Outcomes over Time

**Figure 2:**
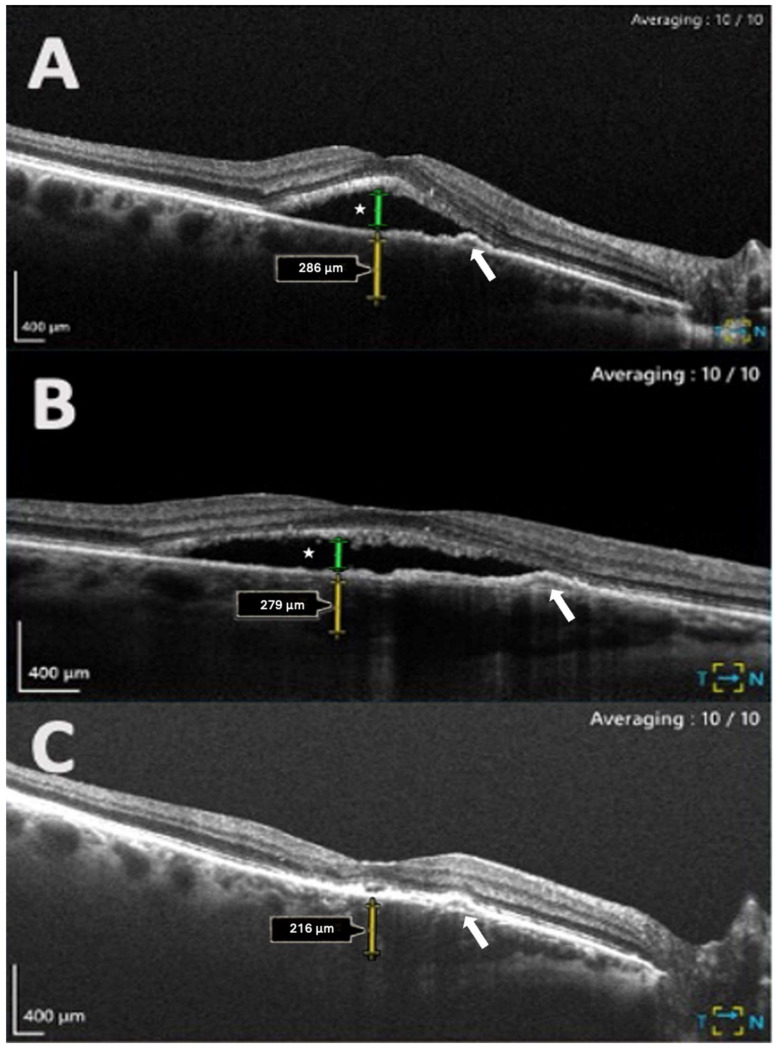
Case of chronic central serous chorioretinopathy (cCSC) complicated by macular neovascularization (MNV) showing response to intravitreal Bevacizumab (IVB) therapy at three time points on spectral domain OCT (SD-OCT) B-scans (**A**–**C**). Subfoveal choroidal thickness measurements are indicated by yellow two-way arrows. (**A**) Baseline B-scan before IVB injections shows a flat irregular pigment epithelial detachment (white arrow) and subretinal fluid (SRF) (SRF height = 374 μm, green two-way arrow). (**B**) B-scan after the first set of three IVB injections demonstrates a reduction in SRF (SRF height = 153 μm, green two-way arrow). (**C**) B-scan at the final follow-up visit shows complete resolution of SRF (SRF height = 0 μm).

Significant reductions in SRF were observed across the total study period, with reductions at each follow-up interval: baseline to post-injection (*p* = 0.007) and baseline to the final follow-up (*p* = 0.003) (Table 2). For CMT, there was no significant reduction across all time intervals using repeated measures ANOVA (*p* = 0.133). However, a significant reduction was observed in the pairwise analysis between baseline and post-injection (*p* = 0.049). All other parameters remained stable during the follow-up period (*p* > 0.05). A representative case showing response to IVB therapy is shown in Figure 2.

**Figure 3:**
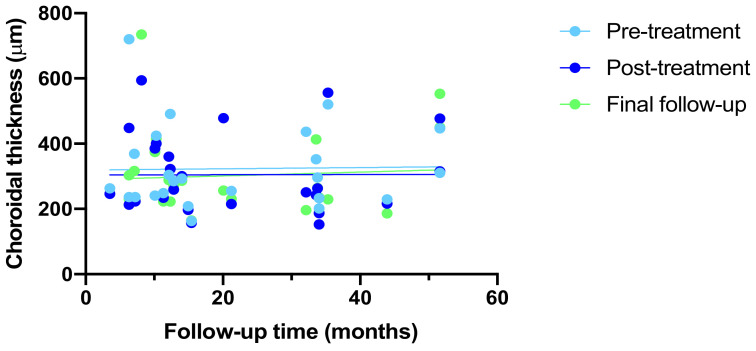
Linear regression analysis of choroidal thickness relative to follow-up time. Choroidal thickness measurements were taken prior to anti-vascular endothelial growth factor (anti-VEGF) injection (pre-treatment, light blue), post-treatment (dark blue), and the final follow-up (green). There was no significant correlation between choroidal thickness and follow-up time at pre-treatment (*p* = 0.9183, r^2^ = 0.000489), post-treatment (*p* = 0.9827, r^2^ = 2 × 10^5^), and final-follow-up (*p* = 0.7628, r^2^ = 0.09341).

Choroidal thickness remained stable across all planes of evaluation (subfoveal (*p* = 0.916), nasal (*p* = 0.970), and temporal (*p* = 0.779)) over time (Supplemental Figure S1). Linear regression analysis confirmed no significant correlation between choroidal thickness and follow-up time: pre-treatment (*p* = 0.9183, r^2^ = 0.000489), post-treatment (*p* = 0.9827, r^2^ = 0.00002), and final follow-up (*p* = 0.7628, r^2^ = 0.09341) (Figure 3). Mixed-effects model analysis also found no significant differences over time, between the groups, or their interactions for these measurements. Greenhouse–Geisser corrections were applied because of violations of the assumption of sphericity (subfoveal: χ^2^(2) = 10.76, *p* = 0.005; nasal: χ^2^(2) = 10.83, *p* = 0.004; SRF: χ^2^(2) = 6.06, *p* = 0.048; CMT: χ^2^(2) = 6.92, *p* = 0.031). Despite these corrections, no significant changes were found in subfoveal, nasal, and temporal CT or in BCVA (logMAR).

### 3.3. Anatomic and Visual Outcomes Relative to Number of Injections

Given the variability in the number of IVB injections received by the patient population (mean 10 ± 8 IVB), The authors assessed choroidal thickness measurements (subfoveal, nasal, and temporal), CMT, and SRF according to the total number of injections received (injection groups: 1–6, 7–12, >12 IVB) across follow-up visits (Table 3). No significant differences in subfoveal, nasal, or temporal CT measurements were observed across the different injection groups at any time point. However, SRF was found to be significantly different between the injection groups at the last follow-up (*p* = 0.047).

Although no significant difference in baseline SFCT was observed between the two groups (responders: 336.75 ± 37.96 µm vs. non-responders: 321.22 ± 27.53 µm, *p* = 0.895), a significant difference in choroidal thickness change by final follow-up was observed between groups. Patients who achieved complete SRF resorption showed an SFCT reduction of 19.13 µm compared to a 29.11 µm increase SFCT by in non-responders (*p* = 0.0294).

### 3.4. Anatomic and Visual Outcomes Between SRF Responders and Non-Responders

**Figure 4:**
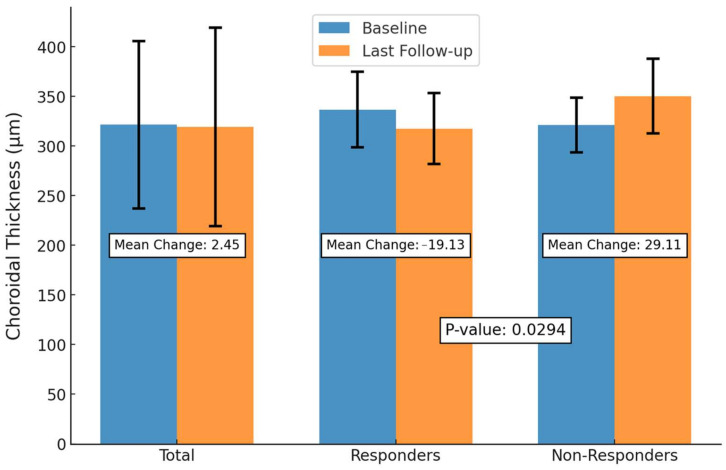
Subfoveal choroidal thickness changes from baseline to last follow-up, stratified by subretinal fluid response. This bar plot shows mean subfoveal choroidal thickness (SFCT) at baseline and last follow-up for responders (complete subretinal fluid [SRF] resorption) and non-responders (persistent SRF), along with the mean change in SFCT between the two time points. Error bars represent standard deviation. Responders exhibited an SFCT reduction of 19.13 µm, while non-responders showed a 29.11 µm increase. The difference in mean SFCT change between the groups was statistically significant (*p* = 0.0294, independent *t*-test).

We also evaluated SFCT changes stratified by those who were SRF responders and those who were non-responders (Figure 4). SRF responders were defined as those who had complete SRF resolution on OCT following three or more IVB injections and at least 1 month between the last injection and OCT. However, one patient received only two injections and achieved a complete response. SRF non-responders included those with persistent subretinal fluid during the follow-up. Nine patients responded to IVB therapy, while nine patients never achieve complete SRF resolution. Notably, four patients who initially responded to SRF experienced SRF recurrence during the study period.

Although no significant difference in baseline SFCT was observed between the two groups (responders: 336.75 ± 37.96 µm vs. non-responders: 321.22 ± 27.53 µm, *p* = 0.895), a significant difference in choroidal thickness change by final follow-up was observed between groups. Patients who achieved complete SRF resorption showed an SFCT reduction of 19.13 µm compared to a 29.11 µm increase in SFCT in non-responders (*p* = 0.0294).

## 4. Discussion

Anti-VEGF injections are the current standard of treatment for patients with MNV secondary to cCSC; however, no treatment algorithm currently exists, largely due to the variable and often incomplete efficacy of anti-VEGF agents in this context. Anti-VEGF agents have been shown to reduce choroidal hyperpermeability and choriocapillaris ischemia in CSC patients [24]. However, substantial controversy exists on whether the choroid responds in a similar manner to anti-VEGF in neovascular CSC compared with AMD. Emerging insights on the pathophysiology of this disease process suggest that SRF may originate from two separate sources; neovascularization induced exudation and CSC disease, which might explain the variability in anti-VEGF treatment efficacy [25]. The variance indicates the challenge to determine which patients would get more benefit either from anti-VEGF treatment, combination therapy, or an alternate regime of treatment.

This study evaluated the visual and anatomic outcomes of intravitreal bevacizumab therapy in patients experiencing neovascular cCSC patients. Our study revealed significant reductions in SRF following treatment, while no significant changes in visual acuity or choroidal thickness were observed. Notably, patient who achieved complete SRF resorption exhibited greater reduction in CT compared to those with persistent fluid, suggesting a differential response among patients.

Although IVB effectively reduced SRF, its impact on other clinical outcomes was limited, as BCVA and CT remained stable across all evaluation planes. Only 40% of patients achieved complete SRF resolution, highlighting the variability in response. Previous studies, such as those by Lejoyeux et al. and Rhomdane et al., have reported similar SRF resorption rates (55% and 45%, respectively) [21,26]. In eyes with persistent fluid, Lejoyeux et al. suggested that SRF was more likely due to CSC rather than choroidal neovascular activity, proposing PDT in these cases [26]. These results underscore the need to differentiate between SRF sources, such as MNV activity versus primary cCSC, to guide treatment decisions accurately.

Unlike other neovascular conditions such as wet AMD, where CT reduction serves as a marker of choroidal hyperpermeability improvement with anti-VEGF treatment, no significant CT changes were observed in our cohort, regardless of injection frequency. This finding aligns with studies by Padron-Perez et al., who found no correlation between CT and injection frequency in similar patient groups [27]. However, some experts have suggested that an extended treatment of anti-VEGF injections may be needed in pachychoroid neovasculopathy patients (CSC with type 1 neovascularization) compared to AMD patients to achieve an optimal effect on choroidal thickening and congestion [28]. Similarly, Schworm et al. reported CT and SRF reductions with extended anti-VEGF treatment, suggesting that pachychoroid neovasculopathy patients might require more intensive therapy compared to AMD patients to achieve optimal choroidal effects [13].

The variability in fluid response may also be influenced by specific choroidal and retinal features. Several parameters, such as choroidal neovascularization (CNV) surface area, flow area [24], serous retinal detachment (SRD), PED height [21,25], pachyvessel diameter, and baseline CT [29], have been identified as potential predictors of anti-VEGF response in cCSC patients. However, the inconsistency observed in studies reporting these variables indicates that even among patients sharing a cCSC diagnosis, there may be phenotypic differences that influence treatment outcomes. For instance, Hagag et al. found no specific clinical or anatomical biomarkers reliably associated with treatment response, emphasizing the heterogeneity of this condition [30].

The variability in fluid response raises questions about the underlying mechanisms influencing SRF reduction with repeated injections. It is possible that the SRF may originate from both neovascular exudation and cCSC pathology, which may explain the variability in treatment outcomes. It has also been suggested that MNV phenotypes can differ substantially in these patients. Carosielli et al. reported more fluid resorption in patients with type 1 neovascularization than mixed type 1 and type 2 [28]. It is possible that our study findings were influenced by our inclusion of patients with mixed type 1 and type 2 MNV, and further research is needed to explore the anti-VEGF responses between these patients. Moreover, the absence of CT changes despite fluid resorption supports the notion that the pathophysiology of MNV in patients with chronic CSC may differ from that described in other neovascular diseases and that MNV may be present without being the primary source of exudation [2,5].

Additionally, visual and anatomic responses might be influenced by the presence of FIPEDs. Multimodal imaging data characterizing clinical features of FIPEDs associated with neovascular cCSC reported by Guo et al. found that vascularized FIPED was closely associated with type 1 CNV and that PDT may be recommended as first line therapy in patients with FIPEDs [31]. Conversely, in a study assessing anti-VEGF levels in patients with FIPEDs with or without CNV, Mao et al. found no significant differences in VEGF levels between those with or without CNV, indicating that anti-VEGF treatment in chronic CSC patients may depend more on the presence of FIPEDs than the presence of neovascularization [32]. Further studies incorporating assessing differences in anti-VEGF between patients with or without vascularized FIPEDs could improve our understanding of the importance of its consideration.

Some reports indicate that different anti-VEGF agents may have varied efficacy in this patient population. Studies like those by Schworm et al. and Chhablani et al. have shown aflibercept to be more effective than other agents such as ranibizumab and bevacizumab [13,33]. However, other studies did not find significant differences in patient outcomes across these agents, suggesting that additional factors may be at play in determining treatment efficacy [28].

It is also important to consider that SRF reduction alone may not be sufficient for visual improvement. Chronicity and severity of the disease, as well as photoreceptor loss and diffuse RPE damage, may limit the potential for visual gain despite anatomical improvements. The presence of chronic disease features such as diffuse RPE irregularity, as seen in our cohort, may partially explain the lack of visual improvement observed. Several studies have evaluated the visual and structural choroidal changes with anti-VEGF monotherapy and combination anti-VEGF + PDT treatment in neovascular CSC. Chen et al. reported that 83% of their patients achieved a fluid-free state, with significant reductions in CRT, SFCT, and CNV vessel density over 3 years of anti-VEGF treatment; however, BCVA remained unchanged [33]. It is noteworthy that most of these patients had received prior treatment and entered the study with MNV in a quiescent state.

The uncertainty in identifying any distinct relationship among SRF, choroidal thickness, and visual outcomes could be attributed to the challenge of distinguishing active MNV from quiescent MNV associated with CSC reactivation. Unlike AMD, choroidal thickness may not correlate with CNV activity in patients with CSC [34,35]. More detailed descriptions of CNV activity following treatment are needed to better predict treatment response.

In regard to choroidal thickness, some studies have reported significant choroidal thinning with anti-VEGF therapy [12,13,21,29], while others have not [25,26]. In our study, the observed differences in choroidal thickness changes between SRF responders and non-responders support the hypothesis that SRF in neovascular CSC may arise from both angiogenesis and/or arteriogenesis. Supporting this, studies that reported choroidal thinning also noted reductions in CNV vessel density and area [12,26], whereas those that did not observe CT changes lacked evidence of CNV remodeling [33,35]. This variability indicates that the response to anti-VEGF therapy is inconsistent and does not always correlate with changes in visual or anatomical parameters [10,26,36,37,38,39].

Despite the efficacy of IVB in reducing SRF, our findings showed no corresponding improvement in visual acuity, even among patients who achieved complete fluid resorption. This discrepancy suggests that SRF alone may not be the primary barrier to visual improvement in these patients. Other factors, such as the natural course of the disease, could play a role; for instance, SRF fluctuation may simply reflect the waxing and waning nature of cCSC [26]. Additionally, the chronicity and severity observed in our patient population likely led to photoreceptor atrophy, similar to what is seen in neovascular AMD and pachychoroid neovasculopathy, thus limiting the potential for visual recovery [40,41]. In our study, all patients exhibited chronic CSC with diffuse RPE irregularity, which could further contribute to the lack of visual improvement. Finally, cytokine concentrations might also play a role in determining the efficacy of treatment for cCSC [1].

This study utilized OCT-A and structural OCT to identify neovascularization and examine the choroidal response to anti-VEGF therapy in chronic CSC with secondary MNV. The OCT-A, which eliminates leakage effects and enables independent segmentation of the retinal and choroidal vascular plexuses, detects CNV more frequently than traditional imaging modalities, addressing the key limitations of dye-based angiography techniques [42,43,44]. Furthermore, our study’s eligibility criteria were more refined than those in other studies, as we included only treatment-naive patients. While this led to some difficulty in comparing our results with those of other studies, we believe that it adds to the validity of our findings.

There are several limitations in this study that warrant consideration. First, the retrospective design and absence of a control group limits our ability to conclusively evaluate treatment efficacy and may introduce biases. Additionally, the small sample size reduces our statistical power to detect significant changes, highlighting the need for larger prospective studies to validate our findings. However, we performed rigorous statistical analysis to ensure that our study had adequate power to determine significant differences despite the small patient population. We did not collect data on the number of RPE alterations per patient, preventing classification of CSC according to newly recommended guidelines by the Central Serous Chorioretinopathy International Group [45]. Furthermore, we did not differentiate between RPE-CSC and choroid-CSC subtypes, missing an opportunity to gain more detailed insights into distinct pathophysiological processes and treatment responses. While we primarily relied on OCTA for diagnosis [34,44,46,47,48,49,50], the absence of routine use of FA and ICGA is acknowledged as a limitation. Variability in treatment timing and injection frequency reflects the absence of standardized protocols for cCSC with secondary MNV, with decisions relying on clinical judgment and patient preferences, as seen in AMD and other neovascular diseases. Despite the inherent variability of retrospective data, our statistical analysis confirmed that these differences did not significantly affect outcomes. By including only treatment-naive patients, we minimized the confounding effects of prior therapies. The potential for spontaneous SRF resolution and individual differences in disease course highlight the inherent complexity of CSC and underscore the need for personalized treatment strategies. Moreover, the potential for SRF to resolve spontaneously, along with barriers such as enduring fibrotic alterations and choroidal venous congestion, may have influenced the CT response. Lastly, the course of CSC can vary among individuals due to genetic predispositions, environmental factors, and lifestyle differences. Although we attempted to address different injection frequencies and limited the influence of diurnal variation, our study may not capture the full spectrum of individual patient experiences.

Future research should focus on larger, long-term prospective studies with control groups to better evaluate the efficacy of anti-VEGF therapy and other treatments in CSC patients with MNV. Furthermore, randomized controlled trials (RCTs) are required to provide robust data for comparing different therapeutic approaches. Exploring the efficacy of combination therapies, such as anti-VEGF with PDT or corticosteroids, could offer more effective management strategies for CSC with MNV. Studies are needed to assess the synergistic effects and optimal treatment protocols for these combinations.

## 5. Conclusions

This study highlights the variability in choroidal and retinal responses to anti-VEGF therapy in patients with chronic CSC and secondary MNV. Although significant reductions in SRF and CMT were observed following treatment, no significant changes in BCVA or choroidal thickness were noted. These findings suggest that the pathophysiology of MNV in CSC may differ from that of other neovascular retinal diseases, necessitating further research to understand the mechanisms driving these responses and to optimize treatment strategies for affected patients. The study also helps to demonstrate the role of evaluating choroidal changes in understanding pathophysiology of MNV in cCSC patients and to further add insights into the role of IVB as a useful therapy.

## Figures and Tables

**Table 1:** Clinical and imaging characteristics of patients at baseline.

Characteristics	
Total no. eyes (patients)	22
Age (years), mean ± SD	67.61 ± 10.65
Sex	
Female, no. (%)	4 (18.00)
Male, no. (%)	18 (82.00)
Eye	
OD, no. (%)	14 (64.00)
OS, no. (%)	8 (36.00)
MNV	
Type 1, no. (%)	11 (50.00)
Mixed type 1 + 2, no. (%)	11 (50.00)
Time from diagnosis to first injection (days), mean ± SD	16.35 ± 13.80
Total follow-up period (months), mean ± SD (IQR)	21.0 ± 14.6 (3.5–51.0)
No. injections, mean ± SD (IQR)	10 ± 8 (2.0–29.0)
BCVA (LogMAR), mean ± SD	0.51 ± 0.47
Anatomic parameters	
SFCT, mean ± SD	321.59 ± 84.32
SRF, mean ± SD	176.86 ± 115.62
CMT, mean ± SD	331.32 ± 130.49
PED, no. (%)	12 (54.55)
FIPED, no. (%)	13 (59.09)
IRF, no. (%)	11 (50.00)

Abbreviations: n: Total Number; SD: Standard Deviation; IQR: Interquartile Range; BCVA; Best-Corrected Visual Acuity: SFCT: Subfoveal Choroidal Thickness; CMT: Central Macular Thickness; SRF: Subretinal Fluid; CT: Choroidal Thickness; FIPED: Flat Irregular PED.

**Table 2:** Anatomic outcomes throughout follow-up.

	Baseline	Post Injection	Last Follow-Up	*p* Value
SFCT, mean ± SD	321.59 ± 84.32	317.45 ± 84.96	319.14 ± 99.96	0.850
Nasal CT, mean ± SD	318.23 ± 84.45	317.09 ± 83.57	319.68 ± 102.81	0.928
Temporal CT, mean ± SD	310.23 ± 80.04	304.50 ± 79.46	308.05 ± 79.07	0.779
SRF, mean ± SD *	176.86 ± 115.62	94.45 ± 83.24	80.95 ± 87.32	**0.003**
CMT, mean ± SD **	331.32 ± 130.49	291.32 ± 106.70	284.77 ± 99.19	0.133
BCVA (LogMAR), mean ± SD	0.51 ± 0.47	0.56 ± 0.50	0.55 ± 0.54	0.336

Abbreviations: No., Total Number; SD, Standard Deviation; SFCT, Subfoveal Choroidal Thickness; BCVA, Best-Corrected Visual Acuity; CMT, Central Macular Thickness; SRF, Subretinal Fluid; CT, Choroidal Thickness. Boldface values indicate significance with a threshold of *p* < 0.05 using repeated measures ANOVA (rmANOVA). Boldface values indicate significance across all three time intervals (baseline, post-injection, and last follow-up) using repeated measures ANOVA (rmANOVA). Pairwise significance between specific time intervals (e.g., baseline vs. post-injection or baseline vs. last follow-up) is denoted with an asterisk (*) for SRF and a double asterisk (**) for CMT. In the pairwise analysis, a significant reduction was observed in SRF (baseline vs. post-injection, *p* = 0.007) and CMT (baseline vs. post-injection, *p* = 0.049).

**Table 3:** CT measurements relative to number of injections.

		1–6 (N = 9)	7–12 (N = 6)	>12 (N = 7)	*p*-Value
**Baseline**	**SFCT (1a)**	341.89 (79.59)	350.83 (111.02)	270.43 (40.49)	0.148
	**Nasal CT (1b)**	338.22 (76.43)	344.67 (119.24)	269.86 (35.98)	0.188
	**Temporal CT (1c)**	324.22 (64.26)	345.67 (115.44)	261.86 (39.16)	0.134
	**SRF (1d)**	159.44 (88.59)	183.00 (151.06)	194.00 (128.33)	0.843
	**CMT (1e)**	366.89 (152.83)	286.67 (111.36)	323.86 (119.00)	0.521
	**VA LogMAR (1f)**	0.58 (0.61)	0.44 (0.17)	0.47 (0.49)	0.854
**Post Injection**	**SFCT (2a)**	335.89 (62.73)	347.17 (127.02)	268.29 (48.54)	0.177
	**Nasal CT (2b)**	337.33 (62.39)	342.00 (126.12)	269.71 (46.30)	0.196
	**Temporal CT (2c)**	316.22 (57.66)	336.33 (122.05)	262.14 (44.02)	0.214
	**SRF (2d)**	108.89 (94.74)	109.67 (87.85)	62.86 (64.37)	0.500
	**CMT (2e)**	315.11 (103.02)	284.33 (87.06)	266.71 (133.32)	0.677
	**VA LogMAR (2f)**	0.59 (0.66)	0.53 (0.30)	0.54 (0.45)	0.973
**Last Follow-up**	**SFCT (3a)**	360.44 (110.28)	324.50 (108.48)	261.43 (49.77)	0.143
	**Nasal CT (3b)**	364.33 (112.93)	321.17 (113.44)	261.00 (47.51)	0.136
	**Temporal CT (3c)**	340.11 (66.31)	317.17 (108.58)	259.00 (41.99)	0.117
	**SRF (3d)**	134.78 (91.10)	41.83 (70.16)	45.29 (64.48)	0.047
	**CMT (3e)**	334.00 (114.85)	257.83 (71.62)	244.57 (79.54)	0.149
	**VA LogMAR (3f)**	0.62 (0.64)	0.43 (0.33)	0.57 (0.59)	0.800

Abbreviations: SFCT, Sub-foveal Choroidal Thickness; CT, Choroidal Thickness; VA, Visual Acuity; SRF, Subretinal Fluid. Values are reported as the mean (standard deviation). CT in the sub-foveal, nasal, and temporal regions relative to the number of injections at different time points (1a, 1b,1c–3a, 3b, and 3c). Sub-foveal choroidal thickness relative to the number of injections for central macular thickness (CMT) and subretinal fluid (SRF) at different time points (1d,1e–3d, 3e). Choroidal thickness, CMT, and SRF measurements were obtained (1) at presentation pre-treatment, (2) post-treatment after the first set of three anti-VEGF injections, and (3) at the final follow-up visit. Patients received a different total number of injections between the pretreatment and final follow-up visits. We observed no differences in values across all parameters and assessment points, given the patient’s total number of injections.

## Data Availability

Data will be made available by corresponding author upon reasonable request.

## Supplementary Materials

The following supporting information can be downloaded at: https://www.mdpi.com/article/10.3390/biomedicines12122760/s1, Figure S1: Choroidal thickness in subfoveal, nasal, and temporal areas relative to follow-up time at different time points; Figure S2: Subfoveal choroidal thickness relative to follow-up time.

## Abbreviations

AMD	Age-Related Macular Degeneration
BCVA	Best-Corrected Visual Acuity
CMT	Central Macular Thickness
CNV	Choroidal Neovascularization
CSC	Central Serous Chorioretinopathy
cCSC	Chronic Central Serous Chorioretinopathy
CT	Choroidal Thickness
EDI	Enhanced Depth Imaging
FA	Fluorescein Angiography
FAF	Fundus Autofluorescence
FIPED	Flat Irregular Pigment Epithelial Detachment
IRF	Intraretinal Fluid
IVB	Intravitreal Bevacizumab
MNV	Macular Neovascularization
OCT	Optical Coherence Tomography
OCTA	Optical Coherence Tomography Angiography
FA	Fluorescein Angiography
PED	Pigment Epithelial Detachment
RPE	Retinal Pigment Epithelium
SRF	Subretinal Fluid
SRD	Serous Retinal Detachment
SFCT	Subfoveal Choroidal Thickness
VEGF	Vascular Endothelial Growth Factor
Anti-VEGF	Anti-Vascular Endothelial Growth Factor
