# Supplementary material for: Choroidal Response to Intravitreal Bevacizumab Injections in Treatment-Naïve Macular Neovascularization Secondary to Chronic Central Serous Chorioretinopathy
Source: Biomedicines. 2024 Dec 3;12(12):2760. doi: 10.3390/biomedicines12122760 (PMC11673265; doi:10.3390/biomedicines12122760)
Supplement: Supplementary file 1 [file biomedicines-12-02760-s001.zip › biomedicines-3309559-supplementary/Supplementary Figure Captions.pdf]

Supplementary Figure Captions

Supplemental Figure 1: Choroidal thickness in subfoveal, nasal, and temporal areas relative to follow-up time at different time points. Choroidal thickness measurements were taken at presentation pre-treatment, post treatment after first set of 3 anti-VEGF injections, and at the final follow-up visit. Follow-up time is the number of years between second and third set of measurements for each case. Across all records, we observe no significant change in choroidal thickness for all planes of evaluation (subfoveal (a), nasal (b), and temporal (c)). One-way repeated measures ANOVA showed no significant differences between choroidal thickness and follow-up time for subfoveal ($p = 0.916$), nasal ($p = 0.970$), or temporal ($p = 0.779$) areas.

Supplemental Figure 2: Subfoveal choroidal thickness relative to follow-up time for central macular thickness (CMT) and sub-retinal fluid (SRF) at different time points. Choroidal thickness, CMT, and SRF measurements were taken (1) at presentation pre-treatment, (2) post-treatment after the first set of 3 anti-VEGF injections, and (3) at the final follow-up visit. Follow-up time is the number of years between second and third set of measurements for each case. One-way repeated measures ANOVA showed no significant differences between choroidal thickness and follow-up time for CMT or SRF ($p = 0.47$ and $p = 0.99$, respectively). This indicates that choroidal thickness in relation to differing CMT and SRF had no significant changes even with the differing lengths of follow-up time. CMT and SRF results were also similar regardless of length of follow-up time.
