# Supplementary figures and images for: Choroidal Response to Intravitreal Bevacizumab Injections in Treatment-Naïve Macular Neovascularization Secondary to Chronic Central Serous Chorioretinopathy
Source: Biomedicines. 2024 Dec 3;12(12):2760. doi: 10.3390/biomedicines12122760 (PMC11673265; doi:10.3390/biomedicines12122760)

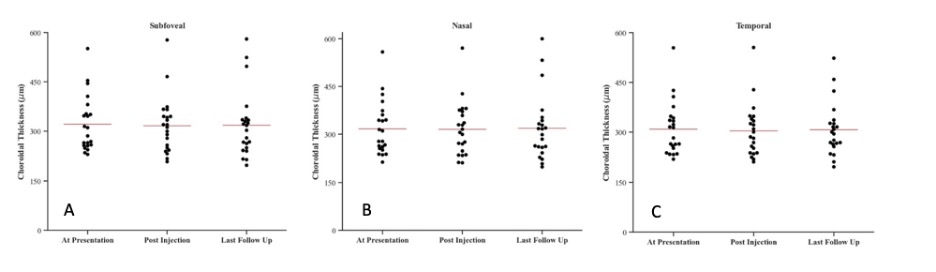

Supplement: Supplementary file 1 [file biomedicines-12-02760-s001.zip › biomedicines-3309559-supplementary/Supplemental Figure 1 (5).jpg]

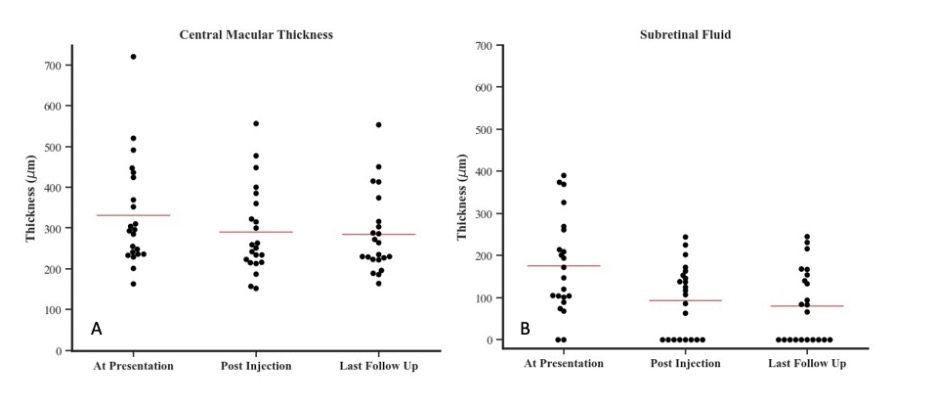

Supplement: Supplementary file 1 [file biomedicines-12-02760-s001.zip › biomedicines-3309559-supplementary/Supplemental Figure 2 (4).jpg]
